# Ultrasound Processing of Vinegar: Modelling the Impact on Bioactives and Other Quality Factors

**DOI:** 10.3390/foods10081703

**Published:** 2021-07-22

**Authors:** Seydi Yıkmış, Filiz Aksu, Sema Sandıkçı Altunatmaz, Başak Gökçe Çöl

**Affiliations:** 1 Department of Food Technology, Tekirdag Namık Kemal University, 59830 Tekirdag, Turkey; 2 Food Processing Department, Faculty of Veterinary Medicine Vocational High School, Istanbul University-Cerrahpaşa, 34000 Istanbul, Turkey; filiz.aksu@iuc.edu.tr (F.A.); sandikci@iuc.edu.tr (S.S.A.); 3 Department of Nutrition and Dietetics, İstanbul Gelisim University, Avcılar, 34000 Itanbul, Turkey; bgcol@gelisim.edu.tr

**Keywords:** response surface methodology, artificial neural network, vinegar, ultrasound, antidiabetic, ACE inhibitor

## Abstract

In recent years, non-thermal technology has been used for the enrichment of ultrasound bioactive components. For this purpose, it was applied to tomato vinegar and modeled with response surface methodology (RSM) and artificial neural network (ANN). At the end of the RSM, cupric reducing antioxidant capacity (68.64%), 1,1-diphenyl-2-picrylhydrazyl (62.47%), total flavonoid content (2.44 mg CE/mL), total phenolic content (12.22 mg GAE/mL), total ascorbic acid content (2.53 mg/100 mL) and total lycopene (5.44 μg/mL) were determined. The ANN model has higher prediction accuracy than RSM. The microstructure, microbiological properties, sensory analysis, ACE (angiotensin-converting–enzyme) inhibitor and antidiabetic effects of the ultrasound-treated tomato vinegar (UTV) (8.9 min and 74.5 amplitude), traditional tomato vinegar (TTV) and pasteurized tomato vinegar (PTV) samples were then evaluated. UTV was generally appreciated by the panelists. It was determined that the microbiological properties were affected by the ultrasound treatment. UTV was found to have more effective ACE inhibitor and antidiabetic properties than other vinegar samples. As a result, the bioactive components of tomato vinegar were enriched with ultrasound treatment and positive effects on health were determined.

## 1. Introduction

Vinegar is produced by fermentation from a variety of carbohydrate-containing substances [1]. It has been used in the composition of foods for many years and has been used in traditional treatments since ancient times. Nutritional content in vinegar includes amino acids, sugars, vitamins, and minerals. Thanks to the functions of these nutrients, in particular bioactive compounds in vinegar organic acids (acetic acid, formic acid, citric acid, malic acid, succinic acids and lactic acid), polyphenols (gallic acid, epicatechin, chlorogenic acid, catechin, coumaric acid and p-caffeic acid), melanidines, carotenoids, phytosterols and tetramethylpyrazine, vinegar has effects on energy supply, regulation of cell metabolism, antioxidation, immunoregulation, anticoagulation, improvement of brain development, antidiabetic effect, regulation of lipid metabolism, liver protection, inhibitory activity, anti-fatigue and antitumor effects [2,3,4,5].

Nowadays, there is growing interest in functional foods based on society’s nutritional awareness. In recent years, it has been observed that the health effects of vinegar have increased popularly among the public. Particularly, studies have reported that vinegar produced by traditional methods may have more effects on health [5,6,7]. Due to the different raw materials and production methods used in vinegar production, their effects on health vary [5,7,8,9]. In most studies, grape and apple vinegar are examined; however, there are limited numbers of studies about the characteristics of different vinegars produced traditionally [2].

The increase in natural products has led to the development of new technologies. Particularly, interest in non-thermal technologies has increased due to the fact that thermal pasteurization technology causes significant decreases in the nutritional value of the product [10]. Interest in innovative non-thermal food preservation methods, especially ultrasound technology, has increased. Ultrasound technology is a successful process application in minimal reductions in bioactive components, food safety of the product and sensory evaluations [11]. Many researchers have found that ultrasound treatment of purple cactus [12], guava juice [13], peach juice [14], strawberry juice [15], sirkencubin syrup [16] and tomato juice [17] caused minimal losses to quality and nutritional value. However, when the literature is searched, it is seen that ultrasound studies are limited in increasing the quality of vinegar [7].

Optimization is used in food processes particularly to minimize the losses of bioactive ingredients. The surface response method (RSM) and artificial neural network (ANN) modeling were preferred in optimization studies. In this study, the aim was to treat tomato vinegar produced by the traditional method for the first time with ultrasound and to optimize the bioactive components (total lycopene, total phenolic content, ascorbic acid content, total flavonoid content, DPPH and CUPRAC) using the RSM and ANN. At the same time, the ACE inhibitor effect, antidiabetic effect, microbiological properties, sensory analysis, and microstructure effects of pasteurized tomato vinegar (PTV), traditional tomato vinegar (TTV), and ultrasound-treated tomato vinegar (UTV) were compared.

## 2. Materials and Methods

The traditional method was used for vinegar production. In the research, the raw material for tomato vinegar production of tomatoes was obtained from Tekirdağ in Turkey with each trial using 10 kg of tomatoes (Rio Grande variant). Tomatoes were first pressed and filtered to obtain the juice. The tomato juice was placed in sterile jars, with each jar inoculated with *Saccharomyces cerevisiae* (Laffort, Bordeaux, France) (3%) to ensure the ethanol fermentation stage. Fermentation lids were closed, and the jars were left at 24–25 °C for 28 days for alcohol fermentation. This fermented product was then transferred into a new sterile jar and inoculated with 10% sharp vinegar as a source of natural acetic acid culture, subsequently maintained at 28 °C for up to 60 days to obtain a low content (0.5% to 1%) of ethanol. Tomato vinegar samples were transferred in 100 mL sterile glass jars. Untreated tomato vinegar was coded (TTV). It was stored at 4 °C until analyzed.

### 2.1. Ultrasound Treatments

The main variables that affect ultrasound treatments are the intensity and frequency of the wave. Amplitude is proportional to the ultrasound intensity, which is the power dissipated per unit surface area (W/cm^2^) of the sonotrode. That is, increasing the amplitude causes an increase in ultrasound power [18]. Different amplitudes were applied in this study. A probe diameter of 10 mm was used in ultrasound treatment. Ultrasound treatments were performed at 26 kHz frequency, 200 W ultrasonic processor (Model UP200St, Hielscher Ultrasonics, Teltow, Germany), at different amplitudes (60%, 65%, 70%, 75% and 80%) and at different times (2, 4, 6, 8 and 10 min) on TTV samples. During ultrasound treatments, an ice water bath was used to keep temperature changes below 40 °C, and temperature changes were measured with a thermometer. Tomato vinegar samples were kept in sterilized 100 mL glass bottles and stored at 4 °C until further analysis. The optimization conditions were defined as ultrasound-treated tomato vinegar (UTV) based on the results.

### 2.2. Pasteurization Procedure 

Pasteurization of tomato vinegar samples was carried out in sterilized 100 mL glass bottles and samples were pasteurized in a water bath (Wisd-Model WUC-D06H, Daihan, Wonju, Korea) at 90 °C for 10 s. Pasteurized tomato vinegar was coded (TTV). It was stored at 4 °C until analyzed.

### 2.3. Modelling Procedure for Response Surface Method

The Response Surface Method (RSM) was used to understand the effect of ultrasound treatment of tomato vinegar on bioactive components. Central Composite Design (CCD) was chosen for RSM. Two factors and 5 levels were determined in the design (Table 1). There are 13 trial points for RSM optimization. Model adequacy, R^2^ and corrected-R^2^ coefficients, lack-of-fit tests and ANOVA results were evaluated. The independent variables were duration (X_1_) and amplitude (X_2_). Dependent variables were total lycopene (TL), total antioxidants (DPPH and CUPRAC), total ascorbic acid (TAC), total phenolic content (TPC) and total flavonoid content (TFC). MINITAB statistical software (Minitab 18.1, Minitab, Inc., State College, PA, USA, 2017) was used for RSM and its graphs were developed with SigmaPlot 12.0 Statistical Analysis Software (Systat Software, Inc., San Jose, CA, USA). The polynomial used by Seydi et al. (2020) was used to create model equations.

### 2.4. Modelling Procedure for Artificial Neural Networks

For ANN, MATLAB Neural Network Toolbox (MATLAB Version R2020b-Mathworks Inc., Natick, MA, USA) was used, which provides an interactive environment for numerical computing, visualization and programming. The Levenberg–Marquardt (LM) combined backpropagation (BP) algorithm was used to create a Feed-Forward neural network (FFNN). The Levenberg–Marquardt algorithm uses 15% of this data as test data, 15% is used as validity transaction data and 70% as training data. MLP (multilayer perceptron) neural network architectures were trained. In ANN modeling, the entire data set was used in calibration for samples evaluated for use in independent five-fold cross-validations. For the prediction of a non-linear relationship between the input parameters (time, amplitude) and response outputs (TL, TAC, TPC, TFC, DPPH and CUPCAC), an artificial neural network was used. The best results were analyzed in different neurons for each response. ANNs were trained using 1000 iterations. The generally used main equation of ANN is shown as below:

(1) $$n_{k}^{h}=\overset{R}{\underset{i=1}{\sum }}w_{kj}^{h}p_{j}+b_{k}^{h},k=1toS$$

where *R* is the number of input variables, *n* is the number of data, *bh* is the bias of the hidden layer, *p* is the input variable, *S* is the number of hidden neurons and *wh* is the weight (Alrugaibah et al., 2021).

To clarify the performance of ANN models, determination coefficient (*R*^2^), root mean square error (RMSE) and absolute average deviation (AAD) were compared between RSM and ANN models. The formulae are written as follows:

(2) $$R^{2}=1-\frac{\sum_{i=1}^{n}{\left(Y_{Predicded}-Y_{Expertmental}\right)}^{2}}{\sum_{i=1}^{n}{\left(Y_{Average}-Y_{Expertmental}\right)}^{2}}$$

(3) $$RSME={\left(\frac{1}{n}\overset{n}{\underset{i=1}{\sum }}{\left(Y_{Predicded}-Y_{Expertmental}\right)}^{2}\right)}^{\frac{1}{2}}$$

(4) $$ADD=\left(\frac{1}{n}\overset{n}{\underset{i=1}{\sum }}\left|\frac{Y_{Predicded}-Y_{Expertmental}}{Y_{Expertmental}}\right|\right)*100$$

where *Y_Expertmental_*, *Y_Predicded_*, *Y_Average_* and *n* are the experimental value, predicted value, average of data and number of the data point, respectively. 

### 2.5. Analysis of Bioactive Compounds

Lycopene concentration was determined with the modified method [19]. The lycopene concentration of each sample was expressed in μg/mL. Calculation of the ascorbic acid concentration in the samples was carried out with AOAC 961.27 vitamin preparation and the ascorbic acid 2, 6 dichlorophenol indophenol-titrimetric method in fruit juices [20]. The results obtained are expressed as milligrams of ascorbic acid per 100 mL sample. Total phenolic content analysis was performed according to the Folin–Ciocalteu method, which is a common method. In the experiment, phenolic substance was determined according to the Folin–Ciocalteu method applied by Singleton and Rossi (1965) [21]. Total phenol content using a gallic acid calibration curve was given as gallic acid equivalent and expressed as milligrams of gallic acid equivalents (mg GAE/mL). Total flavonoid concentrations were calculated colorimetrically by UV spectrophotometer according to the method applied by Zhinsen et al. (1999) [22]. The results were expressed as mg of (+)-catechin equivalent per liter of vinegar sample. DPPH (2,2-diphenyl-1-picrylhydrazyl) free radical scavenging activity was estimated according to the procedure described by Grajeda-Iglesias et al. (2016) with slight modifications [23]. CUPRAC (Cu(II) ion reducing antioxidant capacity) assay was performed according to method recently developed by Apak et al. (2006) [24]. In all assays, absorbance measurements were carried out at 25 °C in a UV-VIS spectrophotometer (SP-UV/VIS-300SRB, Spectrum Instruments, Melbourne, Australia).

### 2.6. Analysis of ACE Inhibitor and Antidiabetic (In Vitro)

The ACE inhibitory activity assay was carried out with some modifications [25]. This method measures the absorbance of hippuric acid from ACE activity from hippuryl-L-histidylL-leucine (HHL). The antidiabetic activity of tomato vinegar (α-glucosidase and α-amylase) was investigated according to the modified method [26]. Acarbose was used as a positive control in antidiabetic analyses. Absorbance measurements were performed by UV-VIS spectrophotometer (SP-UV/VIS-300SRB, Spectrum Instruments, Melbourne, Australia).

### 2.7. Optical Microstructure

For optical microstructure, a small drop of vinegar (~40 µL) was placed onto a microscope slide and crosswise covered with a coverslip. A reflected fluorescence system in an Olympus CX41 light microscope (Olympus, Tokyo, Japan) was used to visually analyze the microstructure of the tomato vinegar. Tomato vinegars were dropped on slides and pictures were taken with a digital camera (Kameram 2.1, Argenit, Istanbul, Turkey) under a magnification of 200X.

### 2.8. Analysis of Microbiological Properties

Serial dilutions of vinegar samples (TTV, PTV and UTV) were performed in peptone water solution for microbial count. Acetic acid bacteria (AAB) counts were determined in Glucose Yeast Extract Calcium Carbonate Agar (GYC, pH 6.8, HiMedia, Mumbai, India). The surface plate method was used and incubated for 5–10 days at 30 °C in plates [27]. PCA (Plate Count Agar—Merck, Darmstadt, Germany) was used for TAPC (total aerobic plate count). Samples were incubated at 28 °C for 48 h. For yeast and mold count, Potato Dextrose Agar (PDA, pH 5.6, Merck, Germany) acidified with 10% tartaric acid (Merck, Darmstadt, Germany) was used. The Petri dishes were incubated at 25 °C for 3–5 days. Total Enterobacteriaceae count was determined in VRBG (Violet Red Bile Glucose Agar-Merck, Darmstadt, Germany) incubated at 37 °C for 24 h. The results were expressed as log colony forming units (CFU) per milliliter of PTV, TTV and UTV [28].

### 2.9. Sensory Analysis

The panelists were asked to assess pungent sensation, general impression, richness in aroma aromatic intensity, taste and ethyl acetate odor of PTV, TTV and UTV samples. They were evaluated by students in the department of gastronomy. A total of 32 female and 23 male panelists evaluated the vinegar samples. TTV, PTV and UTV samples were coded using random letters (CBE, SXY and KMC). Scale scores were: excellent, 9; very good, 8; good, 7; acceptable, 6; and poor, <6. Acceptance of sub-points was accepted as 6. 

### 2.10. Statistical Analysis 

All assays were performed in triplicate and results were expressed as means ± standard deviation (SD). Data were analyzed by performing a one-way analysis of variance (ANOVA) and differences among means were determined using Tukey’s HSD (Honestly Significant Difference) test with a level of significance of *p* < 0.05. Statistical analysis was conducted using SPSS 22.0 software (SPSS Inc., Chicago, IL, USA). Pearson’s correlation coefficients were calculated with the OriginPro version 2020 b (OriginLab, Northampton, MA, USA).

## 3. Results

### 3.1. Total Lycopene and Ascorbic Acid 

Lycopene is abundant in tomatoes, and therefore, the amount in tomato vinegar is also significant. As a result of RSM, the polynomial mathematical equation for the TL value of tomato vinegar samples with time and amplitude factors is given below.

(5) $$\mathrm{Total}\;\mathrm{lycopene}\;\left(\mu g/\mathrm{mL}\right)=2.748+0.0904X_{1}+0.0562X_{2}+0.000318X_{1\;}^{2}-0.000299X_{2}^{2}-0.001000X_{1}X_{2}$$

**Figure 1:**
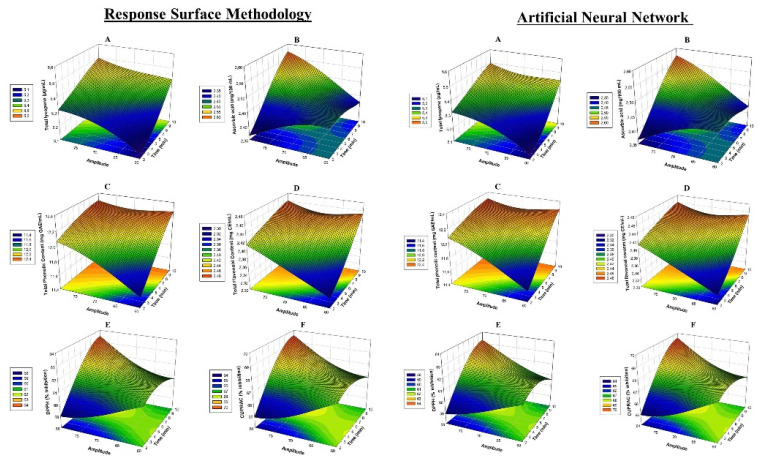
Response surface plots (3D) for TL (**A**), TAC (**B**), TPC (**C**), TFC (**D**), DPPH (**E**) and CUPRAC (**F**) analysis as a function of significant interaction factors for RSM and ANN.

**Figure 2:**
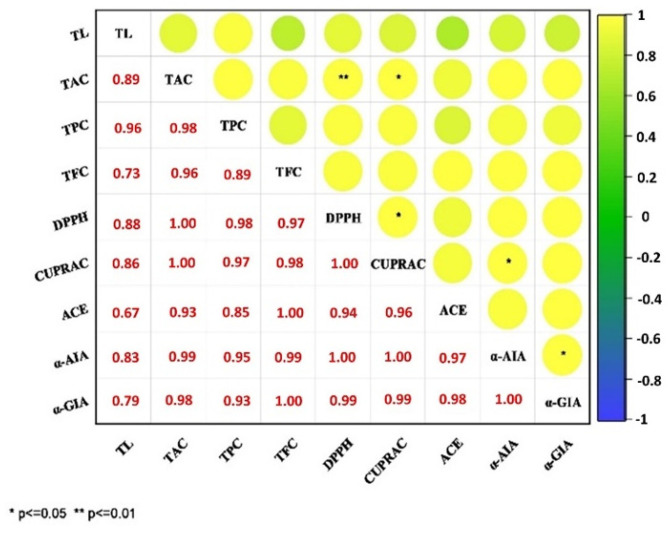
Pearson’s correlation coefficients of TL, TAC, TPC, TFC, DPPH, and ACE inhibitor and antidiabetic of TTV, PTV and UTV. TL: total lycopene; TAC: total ascorbic acid content; TPC: total phenolic content; TFC: total flavonoid content; DDPH: radical scavenging activity; CUPRAC: cupric reducing antioxidant capacity; ACE: ACE Inhibitory Activity %; α-AIA: α-Amylase Inhibitory Activity %; α-GIA: α-Glucosidase Inhibitory Activity %; * *p* ≤ 0.05; ** *p* ≤ 0.01.

It was noted that the coefficient of determination, R^2^, was equal to 97%, indicating the model used in this study to clarify relationships between variables were perfectly fit (Table 2). Linear effects were found to be statistically significant for TL values of X_1_ and X_2_ factors applied to TTV samples (*p* < 0.001). The cross-interactions between time and amplitude variables applied to the TTV were significant (*p* < 0.001). The three-dimensional variation of lycopene content values relative to X_1_ and X_2_ is shown in Figure 1A. Accordingly, a noticeable linear increase in the amounts of TPC was noted in response to time and amplitude applications. The lowest level of TL was 5.23 μg/mL for 6 min in sample number 13 with 60% treatment, and the highest value was 5.44 μg/mL in sample 10 (Table 2). Ultrasound treatment was found to enhance the lycopene values in tomato vinegar; 5.44 μg/mL lycopene was detected after 8.9 min and 74.5 amplitude treatment (Table 3). At the end of the optimization, there was a 4.2% increase in TL compared to the TTV sample. In the pasteurization process, a decrease of 0.4% was found compared to the TTV sample. A high positive correlation was detected between TL and TPC (Figure 2).

A significant increase in total carotenoid levels, total lutein, total β-carotene and total lycopene was reported for cold ultrasound treatment of tomato juice for up to 30 min, peaking at 10 min and progressively decreasing up to 30 min in the next procedure [17]. Enrichment with ultrasound treatment was confirmed by other studies. Lycopene remains relatively resistant to heat-induced oxidation [29]. Therefore, the change in the amount of lycopene in tomato vinegar treated by pasteurization was minimal.

As a result of RSM, the polynomial mathematical equation for ascorbic acid with X_1_ and X_2_ factors of samples is given below.

(6) $$\mathrm{Ascorbic}\;\mathrm{acid}\;\left(\mathrm{mg}/\mathrm{mL}\right)=3.481-0.1543X_{1}-0.01840X_{2}+0.000598X_{1}^{2}+0.000046X_{2\;}^{2}+0.002250X_{1}X_{2}$$

It was noted that the coefficient of determination, R^2^, was equal to 96%, indicating the model used in this study to clarify relationships between variables was perfectly fit. Cross-interactions of factors X_1_ and X_2_ were not significant for TTV samples (*p* > 0.05). Two-way interactions of factors applied to TTV samples are statistically significant (*p* < 0.001). The three-dimensional variation of TAC values relative to X_1_ and X_2_ is shown in Figure 1B. When X_1_ and X_2_ values increased, ascorbic acid amounts generally increased linearly. A statistically positive correlation was found between TAC and DPPH in Figure 2 (*p* ≤ 0.001). The lowest ascorbic acid level was 2.42 mg/100 mL for 7 min and 75% treatment in Example 9; the highest was 2.51 mg/100 mL detected in samples 2 and 8 treated with 75% for 8 min and 70% for 10 min (Table 2). As a result of RSM optimization, it was found that ultrasound treatment of tomato vinegar enriched ascorbic acid values with 2.53 mg/100 mL ascorbic acid detected at the end of 8.9 min and 74.5 amplitude treatment with RSM optimization (Table 3). At the end of the optimization, TAC increased by 4.7% compared to the TTV sample. In the pasteurization process, a decrease of 5.4% was found compared to the TTV sample. As a result of the study, ultrasound-treated strawberry juice displayed an increase in ascorbic acid [15]. The increase in ascorbic acid can be attributed to the removal of dissolved oxygen by cavitation produced during the ultrasound process [13]. Thus, maintaining and/or increasing the amount of ascorbic acid at the end of ultrasound optimization would be a commercial advantage and beneficial for consumer health.

### 3.2. Total Polyphenol Content and Total Flavonoid Content 

As a result of RSM, the polynomial mathematical equations indicating the effect of X_1_ and X_2_ factors on the TPC and TFC values in tomato vinegar samples are given below.

(7) $$\mathrm{TPC}\;\left(\mathrm{mg}\;\mathrm{GAE}/\mathrm{mL}\right)=6.16+0.447X_{1}+0.1147X_{2}-0.00544X_{1}^{2}+0.000520X_{2\;}^{2}-0.00473X_{1}X_{2}$$

(8) $$\mathrm{TFC}\;\left(\mathrm{mg}\;\mathrm{CE}/\mathrm{mL}\right)=0.837+0.0806X_{1}+0.03497X_{2}-0.001546X_{1}^{2}-0.000197X_{2\;}^{2}-0.000750X_{1}X_{2}$$

Linear effects of X_1_ and X_2_ factors applied to tomato vinegar samples for TPC and TFC values were found to be statistically significant (*p* < 0.001). Cross-interactions of X_2_ factors were not significant for TFC (*p* > 0.05). It was determined that the two-way interactions of the factors applied to vinegar were statistically significant for TPC (*p* < 0.05), but not for TFC (*p* > 0.05). The three-dimensional variation of TPC and TFC values relative to X_1_ and X_2_ is shown in Figure 1C,D. It was determined that as the values of X_1_ and X_2_ factors increased, TPC and TFC values were generally enriched. The lowest TPC was 11.87 mg GAE/mL for 2 min and 70% amplitude in Example 4; the highest of 12.24 mg GAE/mL was detected in 75% sample 2 for 8 min. The lowest TFC was 2.37 mg CE/mL for 2 min and 70% treatment in Example 4; the highest was 2.45 mg CE/mL in 75% treated sample 2 for 8 min (Table 2). As a result of RSM optimization, it was determined that the ultrasound treatment of TTV enriched TPC and TFC values. TPC and TFC values were determined as 12.22 mg GAE/mL and 2.44 mg CE/mL, respectively, as a result of RSM optimization at 8.9 X_1_ and 74.5% X_2_ treatment (Table 3). At the end of optimization, TPC and TFC values increased by 3.9% and 2.9%, respectively. Decreases were detected in TTV samples during the pasteurization process.

Bhat et al. (2017) found that there was a significant increase in TPC values compared to the control sample in ultrasound treatments applied to strawberry juice [15]. In a study of apple juice and Kasturi lime juice where ultrasound treatments were applied, they also found an increase in TPC and TFC, and the researchers interpreted this as due to the breakdown of cell walls by the addition of hydroxyl radicals to the aromatic ring of phenolic compounds and the effect of cavitation [11,30]. In the study, as stated in previous reports, the cavitation of micro-shock waves produced by ultrasound treatments caused the cells to break up more and caused TPC and TFC increases.

### 3.3. Antioxidant Activity 

One of the main objectives of this study, thus, was sought to determine the total antioxidant capacity of TTV. For this purpose, the effects of X_1_ and X_2_ on antioxidant contents of TTV based on percentage inhibition of CUPRAC and DPPH activity were determined by using the following equation of the polynomial model of the response surface analysis.

(9) $$\mathrm{DPPH}\;\left(\%\;\mathrm{inhibition}\right)=85.50-2.361X_{1}-0.5164X_{2}-0.05983X_{1}^{2}+0.001777X_{2\;}^{2}-0.04725X_{1}X_{2}$$

(10) $$(\mathrm{CUPRAC}\;\left(\%\;\mathrm{inhibition}\right)=94.72-2.460X_{1}-0.5964X_{2}-0.06351X_{1}^{2}+0.002238X_{2\;}^{2}-0.04950X_{1}X_{2}$$

Linear effects of X_1_ and X_2_ factors applied to tomato vinegar samples for DPPH and CUPRAC values were found to be statistically significant (*p* < 0.001). For DPPH and CUPRAC, cross-interactions of X_1_ and X_2_ factors were statistically significant (*p* < 0.05). The two-way interactions of the factors applied to kinds of vinegar were found to be statistically significant for both antioxidant values (*p* < 0.05). The three-dimensional variation of DPPH and CUPRAC values relative to X_1_ and X_2_ are shown in Figure 1E,F. When X_1_ and X_2_ values increased, it was found that DPPH and CUPRAC amounts generally increased linearly. A statistically positive correlation was found between CUPRAC and DPPH in Figure 2 (*p* ≤ 0.05). The lowest DPPH of 59.76 (% inhibition) was found with 2 min and 70% treatment in sample 4, with the highest of 62.46 (% inhibition) detected in sample 2 with 75% amplitude at 8 min (Table 2). The lowest CUPRAC of 65.75 (% inhibition) was found with 2 min and 70% treatment in sample 4, with the highest rate of 68.56 (% inhibition) with 75% treatment for 8 min (Table 2). At the end of the study, ultrasound-treated tomato vinegar was enriched in total antioxidants. DPPH and CUPRAC values were determined as 12.22 mg GAE/mL and 2.44 mg CE/mL, respectively, as a result of RSM optimization of 8.9 min and 74.5 amplitude treatment (Table 3). At the end of the optimization, DPPH and CUPRAC values increased by 2.9% and 2.8%, respectively. In the pasteurization process, reductions in antioxidants were detected in TTV samples.

It was found that the total amount of antioxidants after treatment with ultrasound applied to purple cactus juice [12], strawberry juice [15] and Kasturi lime juice [30] was enriched compared to control samples. Possible causes of the increase can be attributed directly to ultrasonic-induced cavitation [30]. One of the reasons for the increase in antioxidant amounts in tomato vinegar with ultrasound treatment is from the enrichment of compounds such as ascorbic acid, TPC and TFC.

### 3.4. Comparison between RSM and ANN Models

**Figure 3:**
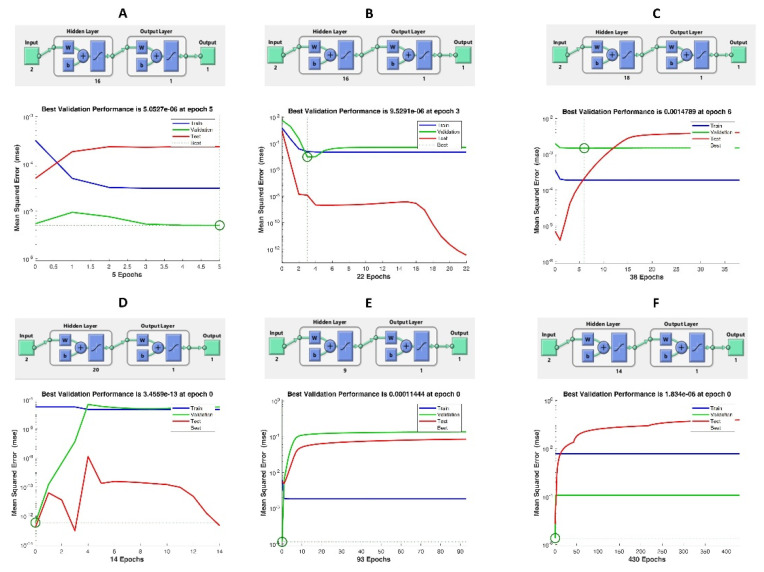
(**A**–**F**) Optimal architecture of developed ANN model and performance plot for the ANN model.

Different numbers of neurons used for responses and best validation values were determined at the end of modeling in Figure 3A–F. When examined in both models, high R^2^ values showed that the predicted and experimental data were suitable (Table 4). R^2^ for RSM (0.97, 0.96, 0.96, 0.96, 0.99 and 0.99 for TL, TAC, TPC, TFC, DPPH and CUPRAC, respectively). For ANN, data for all responses were applied due to their particularly high values for R^2^ (0.999, 0.98, 0.98, 0.98, 0.99 and 0.99 for TL, TAC, TPC, TFC, DPPH and CUPRAC, respectively). Their peak yields predicted by ANN and RSM were close to experimental yields, indicating that ANN is a better approach for predictive modeling than RSM. To compare the RSM and ANN models, the statistical results of the RMSE and ADD parameters are shown in Table 5. As can be seen from the table, R^2^ values of ANN models are higher than RSM modeling. However, models for DPPH antioxidant gave approximate values. In all experimental results, ANN was found to perform better with lower AAD (except DPPH) and RMSE values. RSM is effective as long as it is limited to quadratic polynomial regression. However, ANN’s improved prediction capacity may be related to its universal ability to approach nonlinear systems. ANN is also useful as it is flexible to add and train new experimental data for a model generation [31]. Therefore, it was concluded that the ANN model is more reliable and accurate in terms of predictive ability and compliance with the measured responses compared to the RSM model. Yang et al. 2019, found that the ANN model gave better answers compared to the RSM model in the optimization of kidney bean antioxidants [32]. Similarly, the study comparing the modeling efficiencies for enzyme-assisted (Pectinex) ultrasonic extraction of resveratrol from Polygonum cuspidatum revealed that ANN performed better with higher R^2^ and lower AAD and RMSE values [33]. It has been reported that the ANN model is superior to predict ultrasound-assisted extraction recovery of phenolic compounds of garlic [34]. As a result, ANN modeling for RSM has shown a good alternative and fit.

### 3.5. ACE (Angiotensin-Converting Enzyme)

ACE is important in lowering blood pressure by controlling the overactivation of the angiotensin aldosterone system. Although there are many ACE inhibitor drugs available on the market today, natural ACE inhibitors continue to attract attention. The literature reported the ACE inhibition of flavonoids, especially in plant extracts rich in fruits and apples [35]. As shown in Table 5, the ACE inhibitory activity of the UTV sample is higher than the other samples. The UTV sample was 1.8% higher than the TTV sample and no statistically significant difference was detected (*p* < 0.05). Pasteurization showed the least effect on ACE inhibitory activity with a 25.84 ± 0.79 inhibition effect. In an in vitro study, it was reported that the ACE inhibitory activity of tomato vinegar was between 16–29% with varying degrees of acetic acid content [36]. Thus, it is parallel to this study. In a study of ACE inhibitor rats, when the effects of vinegar were examined, it was found that there were hypertensive effects caused by acetic acid [9]. In this study, the in vitro hypotensive and hypolipidemic effects of ultrasound-treated tomato vinegar were determined. Thus, the results show that tomato vinegar can be used as a functional beverage to control blood pressure and serum cholesterol levels in the body.

### 3.6. Antidiabetic Activity

One of the most effective ways of treating diabetic patients is to control postprandial hyperglycemia. This is effective for regulating/preventing hyperglycemia by inhibiting the hydrolyzing enzymes of carbohydrates in the digestive tract, such as α-amylase and α-glucosidase. Earlier reports stated that many natural products, such as terpenoids, alkaloids, flavonoids and phenolic compounds, can inhibit these enzymes and can assume the expected properties of an antidiabetic agent by delaying carbohydrate absorption [37,38]. In this study, the antidiabetic properties of tomato vinegar (the ability to inhibit α-amylase and α-glucosidase enzymes) were investigated and are presented in Table 5. The α-amylase inhibitory activity of the UTV sample was higher than the TTV sample and no statistically significant difference was detected (*p* < 0.05). When the α-glucosidase inhibitory activity level was examined, the UTV sample caused 2.2% more inhibition than the TTV sample. When we examine the antidiabetic effects of both methods, the PTV sample caused less inhibition than the other samples. In a study of the consumption of apple cider vinegar in patients with type 2 diabetes and dyslipidemia, some evidence was provided that it may have beneficial effects on glycemic indices and oxidative stress [8]. In the literature review of the researchers, the effect of vinegar on blood sugar development was explained in three ways: inhibition of alpha-amylase effect; increased glucose uptake; and transcription factors [4]. The high antidiabetic effect of the ultrasound-treated tomato vinegar sample can be attributed to the enrichment of bioactive components by the effects of cavitation.

### 3.7. Optical Microstructure

**Figure 4:**
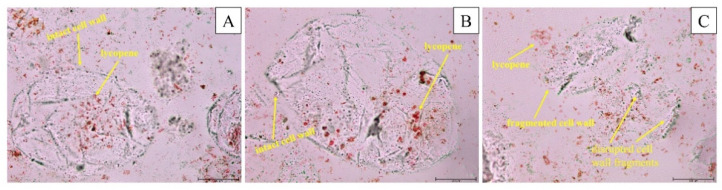
Microstructure of traditional tomato vinegar (**A**), pasteurized tomato vinegar (**B**) and ultrasound-treated tomato vinegar (**C**).

Figure 4 shows microscopic observations of tomato vinegar samples. The cell walls of the TTV and PTV samples are intact. However, in the UTV sample, it was observed that the cell wall was damaged at the parameters determined after optimization. As a result of cell rupture, the surface area of the suspended particles was increased, and the tomato vinegar components were released into the medium. After the application of ultrasound, all the cells that remain intact, the disintegrating tissue, and the parts of the cells remain. The serum phase of tomato vinegar contains water and intracellular-soluble components such as sugars, minerals, and acids. In this sense, the effect of ultrasound treatment may be caused by cavitation and shear [39]. As shown in Figure 4, lycopene is localized within the cell. As seen in ultrasound treatments, cell rupture is evident, and carotenoids and other compounds have been released into the medium. However, some intact cells are still seen due to ultrasound intensity. It has been reported that ultrasound treatment changes the microstructure of strawberry juice [40], peach juice [14] and guava juice [41]. The increase in microstructure degradation can be used in conjunction with ultrasound to explain why the bioactive components increased in this study (Table 2). At the same time, as explained in the report José et al. (2014), disruption, rupture, and leakage of cell structures can be attributed to cavitation effects resulting from ultrasound treatment [42]. 

## 4. Microbiological Quality and Sensory Properties 

Ultrasound treatment is a potential alternative to traditional thermal pasteurization technologies to ensure microbial safety. For this purpose, the microbiological quality of TTV, PTV and UTV vinegar samples was evaluated based on four indicators: AAB, TAPC, TEC and YMC. Microbiological properties of TTV, PTV and UTV samples are given in Table 6. While the number of TPC in the TTV vinegar sample was 2.92 log CUF/mL, it was found to be 1.47 log CUF/mL in the PTV sample (*p* > 0.05). Similarly, the TAPC count of TTV vinegar (3.17 log CUF/mL) was found to be higher (1.29 log CUF/mL) compared to the UTV vinegar sample (*p* < 0.05). However, the YMC numbers of TTV, PTV and UTV vinegar samples were determined as 1.34, 0.85 and 1.13 log CUF/mL, respectively (*p* < 0.05). While the AAB number of the TTV vinegar sample was 4.25 log CUF/mL, it was found to be 1.95 log CUF/mL in the PTV vinegar sample (*p* < 0.05). When TEC numbers were examined, it was not detected in the vinegar samples. It was determined that there was a 0.98 log CUF/mL decrease in the AAB number with ultrasound treatment compared to the TTV sample (*p* < 0.05). The reason for this decrease was thought to be caused by the micro-shock waves created by the cavitation caused by the ultrasound treatment. There is no report on any microbiological quality of tomato vinegar in the literature. Similar results were found by Seydi et al. (2020); they reported that ultrasound treatment affects the microbiological quality of verjuice vinegar [7]. Similar results were observed in microbial changes in different ultrasound treatments applied to the sirkencubin syrup containing vinegar [16]. According to the TTV sample, the decreases in microbial loads in the UTV sample may be caused by cell degradation and physical and chemical mechanisms that occur during cavitation. The results obtained show that the applied processes affect the microbiological quality of the kinds of vinegar.

**Figure 5:**
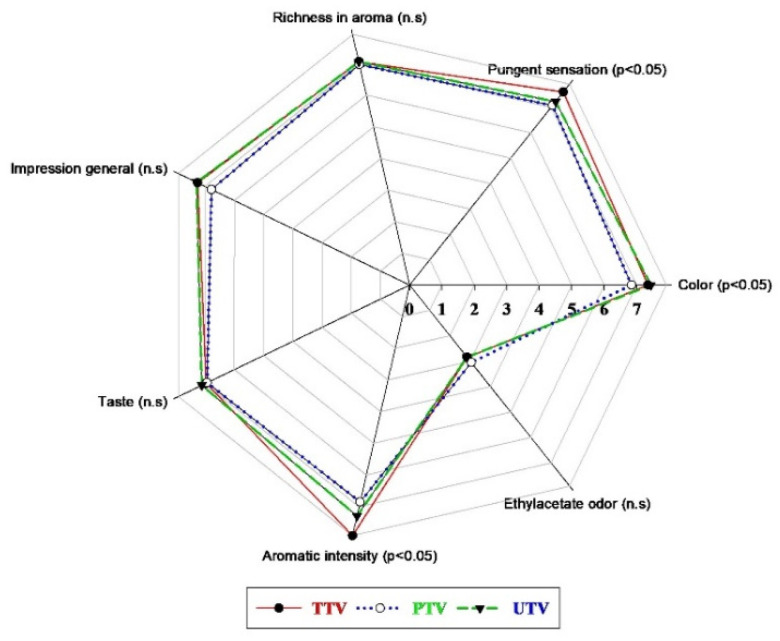
Results of sensory analysis chart for samples. There were statistically significant differences between samples (*p* < 0.05). n.s: no statistical difference; PTV: pasteurized tomato vinegar; TTV: traditional tomato vinegar; UTV: ultrasound-treated tomato vinegar.

Sensory analysis results of TTV, PTV and UTV samples are shown in Figure 5. The UTV sample (7.44) was more liked by the panelists in terms of color compared to the other samples (*p* < 0.05). Panelists preferred PTV (7.07) samples less than other samples in terms of pungent sensation in ultrasound treatment (*p* < 0.05). There was no statistically significant difference between all samples in terms of taste. The TTV (7.91) sample was preferred more than other samples in the evaluation of aromatic intensity (*p* < 0.05). There were no statistically significant differences between TTV (7.28), PTV (6.78) and UTV (7.29) samples in the general evaluation of impression. When the results of the panelists were evaluated, it was determined that the tomato vinegar that was applied with ultrasound treatment was generally liked. Yıkmış et al. (2020) reported ultrasound-treated verjuice vinegar was generally admired by panelists [7]. At the same time, it has been reported that it is generally successful in sensory evaluations as a result of different ultrasound treatments applied to various fruit juices [43,44,45].

## 5. Conclusions

In this study, optimization of process conditions for ultrasound treatment for bioactive components of tomato vinegar was carried out. The microstructure, ACE inhibitor and antidiabetic effects of ultrasound-treated tomato vinegar (8.9 min and 74.5 amplitude), conventional tomato vinegar and pasteurized tomato vinegar samples were also evaluated. Analysis results showed that ultrasound-treated tomato vinegar had enriched bioactive components compared to other samples. The ANN model had a superior prediction ability compared to the RSM model. Treatment of tomato vinegar with ultrasound was generally appreciated by panelists. Tomato vinegar samples treated with ultrasound were found to have more ACE inhibitor and antidiabetic effects than other samples. However, further research on the ACE inhibitor activities, antidiabetic effects and mechanisms of action of tomato vinegar will play an important role in human health and the prevention of diseases. However, further research involving human clinical trials and experimental animal models should support this study.

## Figures and Tables

**Table 1:** Independent variables and their levels in RSM.

Independent Variable	Factor Levels				
	Lowest	Low	Center	High	Highest
	(−1.41)	(−1)	0	(+1)	(1.41)
Time (Factor 1, X_1_)	2	4	6	8	10
Amplitude (Factor 2, X_2_)	60	65	70	75	80

**Table 2:** Analysis of the effects of time and amplitude on bioactive components of TTV by RSM and ANN.

Sample	Encoded Independent Variables		Dependent Variables																	
	X1 (Encoded)	X2 (Encoded)	TL (μg/mL)			TAC (mg/100 mL)			TPC (mg GAE/mL)			TFC (mg CE/mL)			DPPH (% inhibition)			CUPRAC (% inhibition)		
			Experimental Data	RSM Predicted	ANN Predicted	Experimental Data	RSM Predicted	ANN Predicted	Experimental Data	RSM Predicted	ANN Predicted	Experimental Data	RSM Predicted	ANN Predicted	Experimental Data	RSM Predicted	ANN Predicted	Experimental Data	RSM Predicted	ANN Predicted
1	8 (+1)	65 (−1)	5.38	5.36	5.39	2.45	2.45	2.45	12.2	12.19	12.20	2.44	2.43	2.44	61.38	61.29	61.32	67.51	67.43	67.51
2	8 (+1)	75 (+1)	5.43	5.42	5.43	2.51	2.51	2.51	12.24	12.23	12.24	2.45	2.45	2.45	62.46	62.40	62.39	68.56	68.56	68.56
3	6 (0)	70 (0)	5.36	5.35	5.35	2.46	2.46	2.46	12.09	12.14	12.12	2.42	2.43	2.43	61.47	61.58	61.64	67.62	67.71	67.69
4	2 (−1.41)	70 (0)	5.27	5.26	5.27	2.43	2.43	2.43	11.87	11.85	11.86	2.37	2.37	2.37	59.76	59.71	59.79	65.75	65.72	65.75
5	6 (0)	70 (0)	5.34	5.35	5.35	2.46	2.46	2.46	12.14	12.14	12.13	2.43	2.43	2.43	61.65	61.58	61.64	67.82	67.71	67.69
6	6 (0)	70 (0)	5.36	5.35	5.35	2.47	2.46	2.46	12.13	12.14	12.13	2.44	2.43	2.43	61.56	61.58	61.64	67.56	67.71	67.69
7	6 (0)	70 (0)	5.35	5.35	5.35	2.45	2.46	2.46	12.14	12.14	12.13	2.43	2.43	2.43	61.54	61.58	61.64	67.69	67.71	67.69
8	10 (+1.41)	70 (0)	5.44	5.45	5.44	2.51	2.51	2.51	12.23	12.26	12.22	2.44	2.45	2.44	61.47	61.54	61.59	67.62	67.67	67.62
9	4 (−1)	75 (+1)	5.33	5.35	5.33	2.42	2.43	2.42	12.09	12.12	12.09	2.42	2.42	2.42	60.48	60.54	60.49	66.53	66.60	66.53
10	6 (0)	80 (+1.41)	5.41	5.40	5.41	2.48	2.48	2.48	12.22	12.22	12.22	2.44	2.44	2.44	61.92	61.92	61.90	68.11	68.08	68.11
11	4 (−1)	65 (−1)	5.24	5.24	5.24	2.45	2.46	2.45	11.86	11.89	11.86	2.38	2.38	2.38	61.29	61.33	61.29	67.46	67.45	67.46
12	6 (0)	70 (0)	5.34	5.35	5.35	2.46	2.46	2.46	12.18	12.14	12.13	2.43	2.43	2.43	61.65	61.58	61.63	67.82	67.71	67.69
13	6 (0)	60 (−1.41)	5.23	5.24	5.23	2.45	2.45	2.45	11.95	11.95	11.95	2.38	2.38	2.38	61.58	61.60	61.58	67.74	67.79	67.74
PTV			5.19			2.28			11.48			2.14			58.61			64.42		
TTV			5.21			2.41			11.74			2.37			60.66			66.74		

RSM: Response surface methodology; ANN: artificial neural network; TL: total lycopene; TAC: total ascorbic acid content; TPC: total phenolic content; TFC: total flavonoid content; DDPH: radical scavenging activity; CUPRAC: cupric reducing antioxidant capacity; PTV: pasteurized tomato vinegar; TTV: traditional tomato vinegar; GAE: gallic acid equivalent.

**Table 3:** Maximum optimization values according to RSM.

Variable	Setting			
Time (X_1_) (min.)	8.9			
Amplitude (X_2_) (%)	74.5			
Response	Fit	SE Fit	95% CI	95% PI
CUPRAC (% inhibition)	68.64	0.09	(68.4195; 68.8505)	(68.3113; 68.9587)
DPPH (% inhibition)	62.47	0.07	(62.2950; 62.6436)	(62.2075; 62.7311)
TFC (mg CE/mL)	2.44	0.01	(2.43051; 2.45913)	(2.42332; 2.46632)
TPC (mg GAE/mL)	12.22	0.03	(12.1586; 12.2898)	(12.1257; 12.3228)
TAC (mg/100 mL)	2.53	0.01	(2.51582; 2.54454)	(2.50861; 2.55176)
TL (μg/mL)	5.44	0.01	(5.4102; 5.4717)	(5.3947; 5.4871)

TL: Total lycopene; TAC: total ascorbic acid content; TPC: total phenolic content; TFC: total flavonoid content; DDPH: radical scavenging activity; CUPRAC: cupric reducing antioxidant capacity; GAE: gallic acid equivalent.

**Table 4:** Predictive capacity comparison of RSM and ANN models for six response variables.

Parameters	TL (μg/mL)		TAC (mg/100 mL)		TPC (mg GAE/mL)		TFC (mg CE/mL)		DPPH (% İnhibition)		CUPRAC (% İnhibition)	
	RSM	ANN	RSM	ANN	RSM	ANN	RSM	ANN	RSM	ANN	RSM	ANN
R^2^	0.97	0.99	0.96	0.98	0.96	0.98	0.96	0.98	0.99	0.99	0.99	0.99
RMSE	0.011	0.006	0.005	0.004	0.024	0.014	0.005	0.04	0.061	0.073	0.075	0.065
ADD (%)	0.1783	0.0431	0.1531	0.0003	0.152	0.0761	0.169	0.0633	0.0876	0.0863	0.0915	0.0523

R^2^: coefficient of determination; RMSE: root mean square error; AAD: absolute average deviation; RSM: response surface methodology; ANN: artificial neural network; TL: total lycopene; TAC: total ascorbic acid content TPC: total phenolic content; TFC: total flavonoid content; DDPH: radical scavenging activity; CUPRAC: cupric reducing antioxidant capacity; GAE: gallic acid equivalent; CE: catechin equivalent.

**Table 5:** ACE inhibitor and antidiabetic inhibitory activities of PTV, TTV and UTV.

Sample	ACE Inhibitory Activity %	α-Amylase Inhibitory Activity %	α-Glucosidase Inhibitory Activity %
PTV	25.84 ± 0.79 ^a^	41.07 ± 0.71 ^a^	39.80 ± 0.66 ^a^
TTV	28.92 ± 0.66 ^b^	42.09 ± 0.29 ^ab^	41.75 ± 0.76 ^b^
UTV	29.45 ± 0.76 ^b^	42.72 ± 0.43 ^b^	42.68 ± 0.67 ^b^

PTV: pasteurized tomato vinegar; TTV: traditional tomato vinegar; UTV: ultrasound-treated tomato vinegar. Values followed by different letters within the same column are significantly different (*p* < 0.05) (*n* = 3 ± SD).

**Table 6:** Effects of thermal treatment and ultrasound on microbial analysis of tomato vinegar.

Samples		Microbiological Analyzes		
	Acetic Acid Bacteria (Log CFU/mL)	Total *Enterobacteria* Count (Log CFU/mL)	Total Plate Count (log CFU/mL)	Yeast and Mould Count (log CFU/mL)
TTV	4.25 ± 0.11 ^a^	ND	2.92 ± 0.20 ^a^	3.34 ± 0.11 ^a^
PTV	1.95 ± 0.17 ^c^	ND	1.47 ± 0.24 ^b^	0.85 ± 0.09 ^b^
UTV	3.27 ± 0.16 ^b^	ND	1.63 ± 0.19 ^b^	1.13 ± 0.07 ^a^

ND: not detected; CFU: colony-forming unit; PTV: pasteurized tomato vinegar; TTV: traditional tomato vinegar; UTV: ultrasound-treated tomato vinegar. Values followed by different letters within the same column are significantly different (*p* < 0.05) (*n* = 3 ± SD).

## Data Availability

Not applicable.
